# ARIA México 2014: transculturization of a guideline involving 11 national medical societies

**DOI:** 10.1186/1939-4551-8-S1-A286

**Published:** 2015-04-08

**Authors:** Désirée Larenas-Linnemann

**Affiliations:** 1 Hospital Médica Sur, Mexico

## Background

International guidelines on allergic rhinitis (AR) and its impact on asthma (ARIA) have been developed in collaboration with the World Health Organization since 2001, with updates in 2008 and 2010. The ARIA documents have changed the AR classification, improved the diagnosis of co-morbid asthma and have enhanced the treatment of AR.

Although ARIA 2010 has been translated into Spanish, its content is hardly known to physicians outside the circle of experts in Mexico. Mexican specialists felt a formal transculturization of the guideline would enhance its acceptability among primary and specialized health care workers.

## Methods

3 members from 11 professional societies (Allergists, ENTs, pulmonologists, pediatricians and family physicians), each assigned by their presidents, were invited to form part of the Guidelines Developing Group (GDG). The GDG developed a SCOPE document, determining the focus of the guideline; using AGREE-II –an instrument to evaluate quality and adaptability of guidelines- they confirmed ARIA 2010 was of suitable quality and adaptability for Mexico. They then applied ADAPTE, to formally transculturize ARIA 2010 to Mexican reality. A Delphi process was used among GDG members to agree on the Spanish translation of ARIA clinical questions and the exact wording of the replies, formulated into recommendations or suggestions and explaining interpretation of these. Annotations according to Mexican reality (drug safety, costs and cultural issues) were added to the text.

## Results

A total of 45 questions from the original 2010 ARIA were included and divided into six groups covering prevention, medical treatment, immunotherapy and alternative medicine to treat patients with allergic rhinitis with or without asthma. One extra question, not included in the original 2010 ARIA, on the use of Nasal Lavages for AR was created sustained by a systematic literature review. Most of the questions reached agreement in one or two rounds; one question required three rounds.

## Conclusions

When high-quality international guidelines on a certain topic exist, transculturization, using recognized instruments might yield well-sustained documents, of high local value. As such, an easy-to-use, adapted, up-to-date and applicable allergic rhinitis guideline for Mexico is now available.

